# Pregnancy in multiple system atrophy: a case report

**DOI:** 10.1186/1752-1947-5-599

**Published:** 2011-12-30

**Authors:** Lirong Zhu, Nigel J Cairns, Samer D Tabbal, Brad A Racette

**Affiliations:** 1 Department of Neurology, Washington University School of Medicine, St Louis, MO 63110, USA; 2 Department of Pathology & Immunology, Washington University School of Medicine, St Louis, MO 63110, USA

## Abstract

**Introduction:**

Multiple system atrophy is a late, adult-onset α-synucleinopathy with no data on the effect of pregnancy on the disease course. Early stage multiple system atrophy can be difficult to distinguish from Parkinson's disease.

**Case presentation:**

We describe the case of an Irish woman with parkinsonism starting at age 31, initially diagnosed as having dopa-responsive, idiopathic Parkinson's disease, who successfully delivered a full-term child at age 35. Her pregnancy was complicated by severe orthostatic hypotension and motor fluctuations. Two years post-partum, she underwent bilateral subthalamic nuclei deep brain stimulation for intractable motor fluctuations and disabling dyskinesia. After this treatment course she experienced deterioration of motor symptoms and death eight years after disease onset. Post-mortem neuropathological examination revealed striatonigral degeneration and α-synuclein-positive glial cytoplasmic inclusions in brain stem nuclei, basal ganglia and white matter tracts, consistent with a neuropathological diagnosis of multiple system atrophy.

**Conclusions:**

Multiple system atrophy can affect women of child-bearing age and pregnancy may be associated with marked disease progression.

## Introduction

Multiple system atrophy (MSA) is an α-synucleinopathy characterized by akinetic-rigid parkinsonism, autonomic failure, urogenital dysfunction, cerebellar signs, and pyramidal signs in varying combinations [1]. Based on motor presentation, MSA can be classified into two separate but overlapping clinical subtypes: MSA with predominant parkinsonism (MSA-P) and MSA with predominant cerebellar ataxia (MSA-C) [1,2]. Motor features of MSA-P include akinesia/bradykinesia, rigidity, postural instability, and tremor. As many as one-third of patients with MSA-P have rest tremor [3]. According to the 2008 MSA consensus statement, poor levodopa-responsiveness parkinsonism is considered one of the important characteristics to diagnose probable MSA-P [2]. However, the early stage of MSA-P often responds to levodopa and may have subtle or absent autonomic dysfunction, making it difficult to distinguish from Parkinson's disease (PD) [4]. Confirmation of the diagnosis of definite MSA-P is based on neuropathological evidence of Papp-Lantos bodies (α-synuclein-positive glial cytoplasmic inclusions (GCIs)) and striatonigral degeneration [2].

According to the European MSA registry, the mean age at onset is 57.8 years [5]. Given the late-life onset, there is no available information on effects of gestation on the symptoms and course of MSA. Equally important is the effect of MSA and medication treatment on the gestation, fetal development, delivery, lactation and ability to care for a newborn. We describe the case of a woman with pathologically confirmed MSA-P who was diagnosed PD when she became pregnant and underwent a successful pregnancy and delivered a full-term healthy baby.

## Case presentation

A 31-year-old Irish woman who resided in the midwestern USA developed progressive loss of left hand dexterity and rest tremor. Eight months later, she experienced episodes of pre-syncope triggered by going up or down stairs or by a hot environment. Her initial neurological examination revealed rigidity, bradykinesia, decreased left arm swing, and rest/action tremor in the ipsilateral hand but no pyramidal, cerebellar signs or objective orthostatic hypotension signs. Unified Parkinson's Disease Rating Scale motor subsection part III (UPDRS III) score [6] was 18 points. Results of routine blood examination, ceruloplasmin, and a cerebral magnetic resonance imaging (MRI) scan were normal. She was diagnosed as having early-onset idiopathic Parkinson's disease, based upon gradual onset asymmetric parkinsonism with rest tremor. She initially declined pharmacological treatment until her symptoms spread to her left lower extremity and right hand one year later. She was started on a dopamine agonist pramipexole 0.75 mg three times a day, with good results.

Six months later, she and her husband decided to have a child. We advised her and her husband that carbidopa/levodopa had been reported to be used safely in pregnant women with PD [7,8], but there was insufficient information about the safety of pramipexole in pregnancy. As a result, pramipexole was switched to levodopa/carbidopa. She had excellent response to levodopa/carbidopa with mild levodopa-induced dyskinesias at doses that provided optimal motor benefit, 900 mg levodopa/day. Five months after she started taking levodopa, her UPDRS III score decreased from 20.5 to 11 points and she became pregnant with her first child.

During her second month of pregnancy, she developed syncope after arising from a supine position. She noticed that she had less responsiveness to levodopa and her ON state total UPDRS score was 48 points with UPDRS III subscore of 30 points. She had marked symptomatic orthostatic hypotension with supine blood pressure 116/70 mmHg and standing blood pressure 70/50 mmHg. Her motor function worsened substantially, and by pregnancy week 30, her total UPDRS score was 56.5 points with UPDRS III subscore of 38.5 points. The dose of levodopa was not increased throughout her pregnancy due to potential exacerbation of orthostatic hypotension.

At age of 35, she gave birth to a full-term 2.9 kg girl with Apgar scores of eight at one minute and nine at five minutes by caesarean section, due to breech presentation. General and neurological examination of the baby revealed no abnormalities and the routine neonatal blood tests were normal. She did not breastfeed her child. At age five years, her daughter had developed normally except for recurrent strabismus and a dermoid cyst in the anterior neck. Given that MSA is usually considered to be a rare and sporadic disease [1], her daughter was not followed by a neurologist routinely.

One week after delivery, her total UPDRS score decreased to 37 points with UPDRS III subscore of 22 points on the same levodopa dose. Her blood pressure was 110/60 mmHg when lying and 100/62 mmHg when standing. Despite symptom improvement in the first post-partum month, she had subsequent worsening (with a UPDRS score of 60.5) necessitating escalation of levodopa. Entacapone was added after her orthostatic hypotension was improved with midodrine. She had good initial response, but developed marked motor fluctuations with increase OFF time, peak-dose/diphasic dyskinesias and orthostatic hypotension, despite multiple medication adjustments.

At age of 37, she received bilateral subthalamic nuclei deep brain stimulation (DBS) surgery uneventfully after an in-patient motor evaluation showing response to levodopa (OFF and ON states with UPDRS III subscore 62 and 20.5, respectively). A pre-surgical brain MRI scan revealed no atrophy or abnormal signal in the putamen or hot cross bun sign in the pons. However, she had very limited and transient benefit from subthalamic nuclei deep brain stimulation (STN-DBS) therapy despite multiple attempts of programming. She developed urinary incontinence, progressive dysarthria, dysphagia, and inhalatory/exhalatory stridor within a few months. Her dysarthria and dysphagia were worse after taking her medications, but she was still able to take food by mouth. Within a year, she required a wheelchair, assistance with all activities of daily living, an indwelling urinary catheter for urinary retention, and an augmentative communication device for levodopa-induced dysarthria. Her body mass index (BMI) was 20.5 two months prior to her death, compared to 23 when she was diagnosed PD. All these symptoms raised the possibility of MSA-P. Nearly eight years into her disease course, she died in her sleep possibly due to the laryngeal stridor.

**Figure 1:**
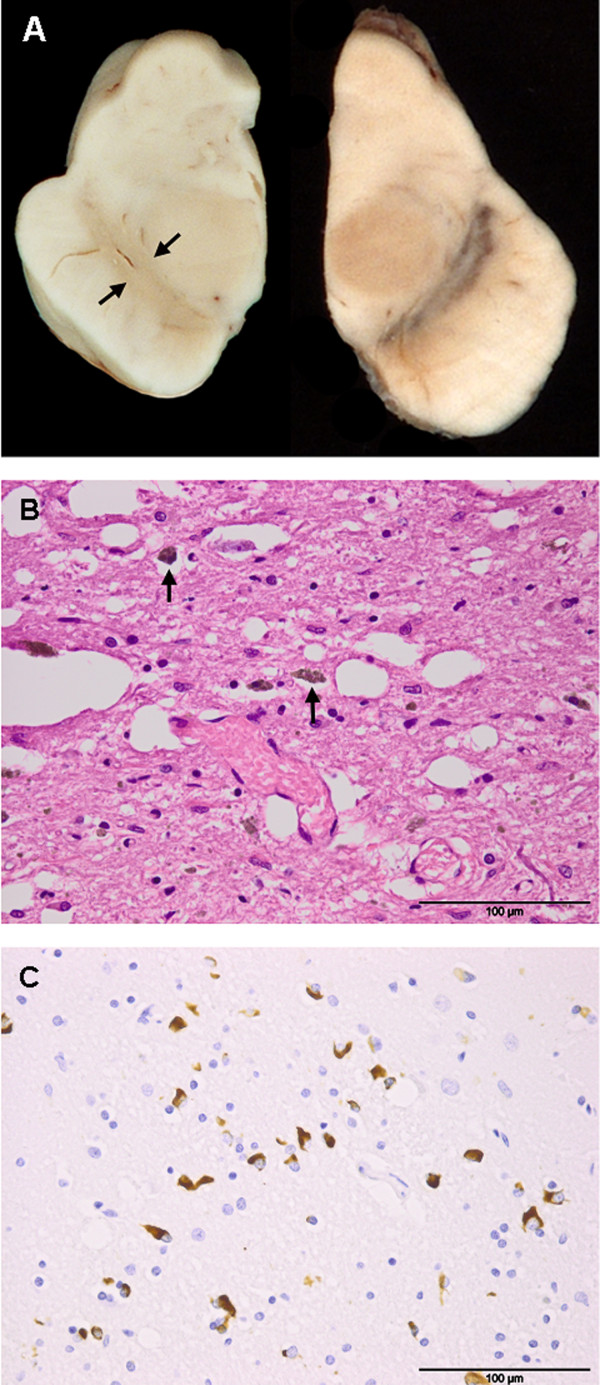
**(A-C) Macroscopic and microscopic features of our patient's brain (post-mortem)**. (A) Left: hemi-midbrain showing pallor of the substantia nigra (between arrows); right: control midbrain showing normal pigmentation. (B) Severe neuronal loss, microvacuolation, and gliosis in the substantia nigra of affected hemi-midbrain. Macrophages containing neuromelanin pigment (arrows). (C) Numerous glial cytoplasmic inclusions in the internal capsule. Stain types: (B), hematoxylin and eosin; (C), α-synuclein immunohistochemistry. Bars: 100 μm.

**Figure 2:**
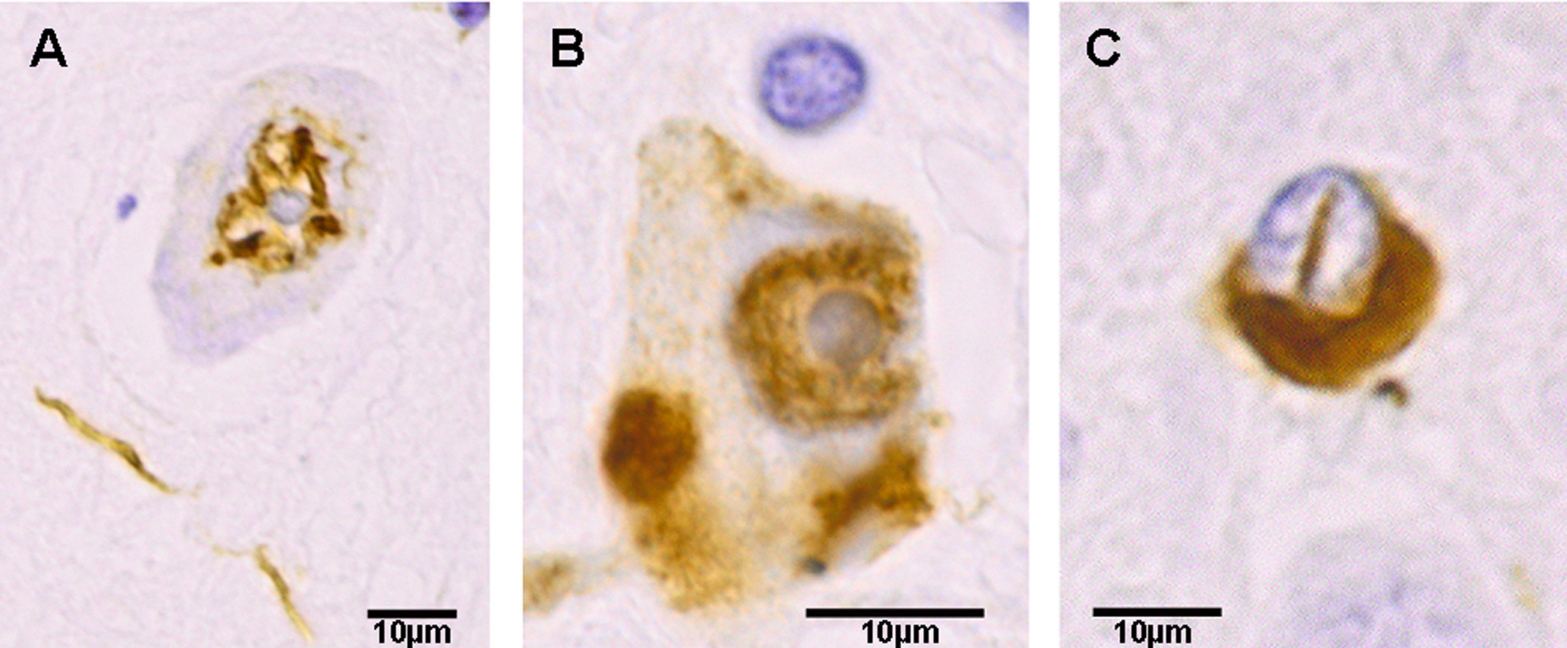
**(A-C) α-Synucleinopathy**. (A) Neuronal intra-nuclear inclusion and dystrophic neuritis. (B) Neuron in the pontine nuclei with intra-nuclear and pleomorphic neuronal cytoplasmic inclusions. (C) Intra-nuclear and cytoplasmic inclusions in oligodendrocytes. Stain types: (A-C), α-synuclein immunohistochemistry. Bars: 10 μm.

Post-mortem macroscopic examination revealed markedly depigmented substantia nigra and locus coeruleus (Figure 1A), and mildly atrophic brainstem and cerebellum. The two stimulation electrodes terminated in the subthalamic nuclei. Microscopic examination showed severe loss of neurons, gliosis, and extracellular pigment in the substantia nigra (Figure 1B) and locus coeruleus; there was also gliosis and neuronal loss in the basis pontis, medulla oblongata, and cerebellum. There were numerous α-synuclein-immunoreactive glial cytoplasmic inclusions (GCIs), Papp-Lantos bodies [9] in the substantia nigra (Figure 1C), red nucleus, crus cerebri, striatum, pallidum, medulla oblongata, and white matter of the frontal lobe and cerebellum. Fewer GCIs were seen in the white matter of the temporal and parietal lobes, hippocampus, thalamus and subthalamic nucleus. There were numerous GCIs, few glial intra-nuclear inclusions (GIIs), dystrophic neurites (DNs), and scattered neuronal intra-nuclear inclusions (NIIs) and few neuronal cytoplasmic inclusions (NCIs) throughout the pontine nuclei (Figure 2A-C). Although there was no macroscopic atrophy noticed in the putamen, minimal neuronal loss, mild gliosis and numerous α-synuclein-immunoreactive GCIs were observed. No tau-positive pre-tangles or α-synuclein immunoreactive Lewy bodies were seen. These pathological features combined with her clinical presentation led to a diagnosis of MSA-P.

## Discussion

To the best of our knowledge, this is the first reported case of a successful pregnancy in a woman with pathologically proven MSA. Her pregnancy was complicated by objective evidence of substantial neurological decline, which may have been due to natural disease progression. However, her clinical symptoms transiently improved shortly after the delivery, suggesting a specific effect of pregnancy on her symptoms. Similar observations have been reported in pregnant women with PD [8,10]. The mechanism underlying the increase of motor disability during pregnancy is poorly understood. Pharmacokinetic variation of levodopa caused by the volume and metabolic changes of pregnancy could be a potential explanation. Other possible mechanisms include changes in hormone levels such as estrogen, suggested by the positive and negative effect of estrogen on central dopaminergic activity [11,12]. Pregnancy can exacerbate orthostatic hypotension, even in the setting of normal autonomic function, due to the expansion of circulatory system volume.

Most medications used for PD and orthostatic hypotension are considered pregnancy category C based on animal data but lack of adequate human evidence of teratogenicity [10]. Levodopa/carbidopa is generally considered to be safe in pregnancy given multiple successful case reports of healthy deliveries in women with PD [10]. Our patient used only levodopa and carbidopa during her pregnancy to avoid less studied effects of dopamine agonists on fetal development. A relationship between the use of levodopa-carbidopa and strabismus or dermoid cyst has never been reported and is unknown.

The early stage of MSA can be difficult to distinguish from Parkinson's disease [4]. Our case confirms that DBS is largely ineffective in MSA, as previously reported [13]. Future studies to develop early diagnostic biomarkers to distinguish MSA from PD are crucial. Sporadic, early-onset PD cases often have homozygous Parkin mutations [14]. Although our patient has no family history of PD or MSA, we speculate that genetic factors may have influenced the onset of MSA at an unusual early age. Genome-wide association studies of patients with MSA have found genetic variants within the SNCA locus are associated with increased risk for development of MSA [15]. Further study will be needed to confirm if mutant α-synuclein was responsible for our patient's early-onset disease.

## Conclusions

MSA can affect women of childbearing age and pregnancy may be associated with marked disease progression.

## Consent

Written informed consent was obtained from the patient's next-of-kin for publication of this case report and any accompanying images. A copy of the written consent is available for review by the Editor-in-Chief of this journal.

## Competing interests

The authors declare that they have no competing interests.

## Authors' contributions

LZ analyzed and interpreted the data from our patient and wrote the first draft. SDT and BAR were involved in the care of our patient. NJC was involved in post-mortem macroscopic and microscopic examinations. LZ, BAR, NJC and SDT participated in discussions and assisted in revising the report. All authors read and approved the final version of the manuscript.
